# Relationship between Eccentric-Exercise-Induced Loss in Muscle Function to Muscle Soreness and Tissue Hardness

**DOI:** 10.3390/healthcare10010096

**Published:** 2022-01-04

**Authors:** Andreas Konrad, Kazuki Kasahara, Riku Yoshida, Kaoru Yahata, Shigeru Sato, Yuta Murakami, Kodai Aizawa, Masatoshi Nakamura

**Affiliations:** 1 Institute of Human Movement Science, Sport and Health, Graz University, Mozartgasse 14, 8010 Graz, Austria; 2 Department of Physical Therapy, Faculty of Rehabilitation, Niigata University of Health and Welfare, 1398 Shimamicho, Kitaku, Niigata 950-3198, Japan; rpa18029@nuhw.ac.jp (K.K.); rpa18121@nuhw.ac.jp (Y.M.); rpa18001@nuhw.ac.jp (K.A.); 3 Institute for Human Movement and Medical Sciences, Niigata University of Health and Welfare, 1398 Shimamicho, Kitaku, Niigata 950-3198, Japan; hpm21017@nuhw.ac.jp (R.Y.); hpm20011@nuhw.ac.jp (K.Y.); hpm19006@nuhw.ac.jp (S.S.)

**Keywords:** muscle strength, range of motion, muscle damage, pain-pressure threshold

## Abstract

It is well-known that unusual exercise, especially eccentric contraction (ECC), could cause delayed-onset muscle soreness. However, the factors related to the loss of muscle strength and range of motion (ROM) caused by eccentrically damaged muscle, such as increases in muscle soreness, tissue hardness, and pain threshold, have not been investigated in detail. Thus, this study was conducted to investigate the factors related to the loss of muscle strength and ROM caused by eccentrically damaged muscle in a large sample. Fifty-six sedentary healthy young male volunteers were instructed to perform 60 repetitions of ECC exercise. The outcome variables were measured before and 48 h after the ECC exercise. The results showed that a decrease in ROM was correlated to an increase in tissue hardness, whereas a decrease in muscle strength was correlated to an increase in muscle soreness. Our results suggested that tissue hardness must be controlled for ROM loss, and muscle soreness must be controlled for muscle-strength loss.

## 1. Introduction

It is well-known that unusual exercise, especially eccentric contraction (ECC), could cause muscle damage. It is a condition characterized by transient ultrastructural myofibrillar disruption, muscle-strength loss, delayed-onset muscle soreness (DOMS), swelling, reduced range of motion (ROM), systemic efflux of myocellular enzymes, and proteins [1,2]. The time course of changes in muscle strength ROM, DOMS, swelling, and blood creatine kinase (CK) in the day after intense eccentric exercise varies, and the loss of muscle strength and ROM and increase in muscle soreness could peak 24–48 h after intense ECC exercise [1]. Additionally, the ECC emphasized that resistance training could cause larger muscle-strength increase and muscle hypertrophy [3,4,5]. Since such impairments after intense ECC exercise could influence athletic performance, reduce training quality, reduce adherence to resistance training, and result in a higher injury prevalence, it is necessary to control the loss of muscle strength and ROM and increase muscle soreness after intense ECC exercise.

Previous studies investigated the methods for estimating the degree of muscle damage. For example, Nosaka et al. (2006) suggested that maximal voluntary isometric contraction (MVC-ISO) loss after ECC exercise does not correlate strongly with changes in markers of muscle damage (i.e., ROM, swelling, and muscle soreness) [6]. However, Dames et al. (2016) investigated the response of markers (i.e., ROM, swelling, CK activity, and muscle soreness) among a large number of young men (*N* = 286). They suggested that MVC-ISO loss could reflect the indirect maker for muscle damage [7]. Additionally, the rate of force development (RFD) could be a more sensitive indirect marker of muscle damage [8,9]. Hence, the changes in MVC-ISO and RFD after ECC exercise are sensitive indirect markers for muscle damage induced by ECC exercise.

The factors related to the loss of muscle strength and ROM caused by eccentrically damaged muscle, such as increases in muscle soreness, tissue hardness, and pain threshold, have not been investigated in detail. Specifically, the factors related to the loss of muscle strength and ROM occurring in the 2 days after ECC exercise, when muscle soreness reaches its peak, are unknown. Thus, this study was conducted to investigate the factors related to the loss of muscle strength and ROM caused by eccentrically damaged muscle. If muscle soreness is associated with ECC-exercise-induced loss in muscle function, thermal agent intervention [10] and/or foam rolling intervention might be effective for improvements [11,12,13]. Moreover, stretching intervention could be an effective approach if tissue-hardness increase is associated with ECC-exercise-induced loss in muscle function [14]. Hence, this study was aimed to determine the factors associated with loss of muscle strength and ROM after ECC exercise in a large sample. We hypothesized that the loss of muscle strength and ROM after ECC exercise could be related to an increase in muscle soreness.

## 2. Materials and Methods

### 2.1. Experimental Design

**Figure 1:**
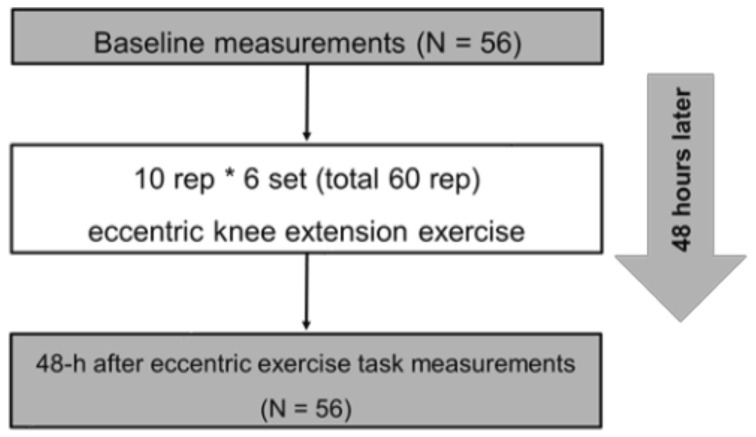
Experimental protocol.

The outcome measurements consisted of knee flexion ROM, MVC-ISO, maximal voluntary concentric contraction (MVC-CON) torque of knee extensor, tissue hardness, pain pressure threshold (PPT), muscle soreness at MVC-ISO, MVC-CON, and stretching before maximal eccentric contraction task (baseline) and 48 h after ECC exercises task (Figure 1). Moreover, we calculated the changes (Δ) from baseline to 48 h after the ECC exercise task. All participants performed 60 repetitions of eccentric (ECC) exercise (10 repetitions * 6 sets) of the knee extensors with the dominant leg (preferred leg for kicking a ball) after baseline measurement. All measurements were taken for each participant at the same time between days. Additionally, the participants became familiarized with all measurements and ECC exercises before baseline measurement in the measurement leg (dominant leg).

### 2.2. Participants

Fifty-six sedentary healthy young male volunteers (age, 21.0 ± 0.9 years; height, 172.1 ± 5.9 cm; body mass, 65.3 ± 8.5 kg) who had not performed habitual exercise activities or had not been involved in any regular resistance training or flexibility training at least for the past 6 months before the measurements participated in the study. We excluded participants who had a history of neuromuscular disease or musculoskeletal injury on the lower extremity. All subjects were fully informed of the procedures and purpose of the study, and all gave written informed consent. The study was approved by the Ethics Committee at the Niigata University of Health and Welfare, Niigata, Japan.

G*Power software (v 3.0.10; Dr. Franz Faul, Kiel University, Kiel, Germany) was used to calculate the sample size on the effect size for correlation analysis (effect size = 0.5 (large), α = 0.05 and power = 0.95), and the elicited results suggested that the minimum required subjects’ number was 42 for this study.

### 2.3. Procedures

#### 2.3.1. MVC-ISO and MVC-CON

Using a dynamometer, MVC-ISO was measured at two different angles, namely, 20° and 70° knee angles. After three submaximal isometric contractions as a warm-up, the participants were instructed to perform maximal contraction of the knee extensors for 3 s at each angle, two times, with a 60 s rest between trials. The average value was adopted for further analysis. MVC-CON was measured at an angular velocity of 60°/s for the ROM of 70° (20–90° knee angles) for three continuous MVC-CONs for the extension after two or three submaximal isometric contractions as a warm-up. The highest value among the three trials was adopted for further analysis. Verbal encouragement was provided consistently during all tests.

#### 2.3.2. Knee Flexion ROM

Each participant was placed in a side-lying position on a massage bed, and the hip and knee of the nondominant leg were flexed at 90° to prevent pelvis movement during ROM measurements [11]. Next, the investigator brought the dominant leg to full knee flexion with the hip joint in a neutral position. Finally, a goniometer was used to measure the knee flexion ROM three times, and the average value was used for further analysis.

#### 2.3.3. Muscle Soreness

Using a visual analog scale that had a continuous line 100 mm with “not sore at all” on one side (0 mm) and “very, very sore” on the other side (100 mm), the magnitude of knee-extensor muscle soreness was assessed by muscle contraction, stretching, and palpation [11,15]. Both MVC-ISO and MVC-CON assessed muscle soreness on contraction, and the average value was adopted for further analysis. For muscle soreness during palpation, participants lay supine on a massage bed, and the investigator palpated the proximal, middle, and distal points of the vastus medialis, vastus lateralis, and rectus femoris [11,16]. The muscle soreness at palpation was measured by a physical therapist with more than 10 years of experience. Again, the average value of the knee extensor palpation points was used for further analysis. As for muscle soreness during stretching, ROM measurement was measured three times, and the average value was used for further analysis.

#### 2.3.4. PPT

An algometer measured PPT measurements (NEUTONE TAM-22 (BT10); TRY ALL Corp., Chiba, Japan) in the supine position. The measurement position was set at the midway of the distance between the anterior superior iliac spine and the upper end of the patella of the dominant side for the rectus femoris muscle. With continuously increasing pressure, the metal rod of the algometer was used to compress the soft tissue in the measurement area. Participants were instructed to immediately press a trigger when the pain was experienced rather than just pressure. The value read from the device at this time point (kilograms per square centimeter) corresponded to the PPT. Based on previous studies [17,18], the mean value (kilograms per square centimeter) of the three repeated measurements was taken with a 30 s interval for data analysis.

#### 2.3.5. Tissue Hardness

Tissue hardness was measured by using a portable tissue hardness meter (NEUTONE TDM-Z2; TRY-ALL Corp., Chiba, Japan). The measurement position and the posture of the participant were similar to PPT measurement for the rectus femoris. All participants were instructed to relax during tissue hardness measurements. Tissue-hardness measurements were repeated thrice, and the average value was used for further analysis.

#### 2.3.6. ECC Exercise Task

All participants performed six sets out of 10 maximal ECC of the unilateral knee extensors (dominant leg) on an isokinetic dynamometer (Biodex System 3.0, Biodex Medical Systems Inc., Shirley, NY, USA) [11]. Participants sat on the dynamometer chair at an 80° hip flexion angle, with adjusted Velcro straps fixed over the trunk, pelvis, and thigh of the exercised limb. The participants were instructed to perform the maximal ECC from a slightly flexed position (20°) to a flexed position (110°) at an angular velocity of 60°/s [11]. After each ECC, the lever arm passively returned the knee joint to the starting position at 10°/s, which gave a 9 s rest between contractions. After 2 or 3 submaximal ECC contractions as familiarization trials, each set was repeated 10 times, and a 100 s rest was given between sets to complete the six sets. The participants received strong verbal encouragement during each ECC to generate maximum force.

### 2.4. Test–Retest Reliability of the Measurements

Test–retest reliability was assessed by the coefficient variation (CV) and the intraclass correlation coefficient (ICC), using 6 healthy men (23.8 ± 4.9 years, 169.4 ± 5.0 cm, 75.7 ± 15.4 kg), with 2 days between the two measures, without any intervention. The CV and ICC of the measurements are shown in Table 1. The ICC ranged from 0.733 to 0.987, and CV ranged from 0.6 to 7.9%.

### 2.5. Statistical Analysis

SPSS (version 24.0; SPSS Japan Inc., Tokyo, Japan) was used for statistical analysis. The data distribution was assessed by using the Shapiro–Wilk test, and we confirmed that the data followed a normal distribution. We calculated the changes (Δ) from baseline to 48 h after the ECC exercise task, and Pearson’s product–moment correlation coefficient was used to quantify the relationship between Δknee flexion ROM, ΔMVC-ISO, ΔMVC-CON and Δmuscle soreness, ΔPPT, and Δtissue hardness. Data are presented as mean ± SD.

## 3. Results

**Figure 2:**
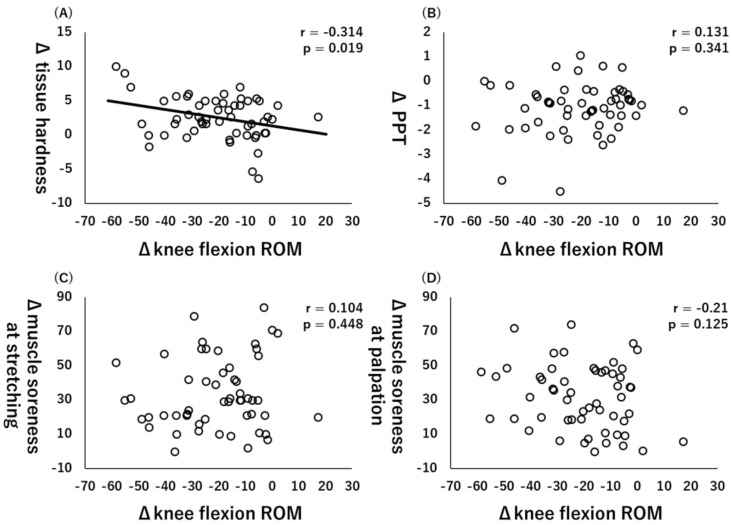
Relationships (Pearson r and *p*-values) between Δknee flexion range of motion (ROM) and Δtissue hardness (**A**), Δpain pressure threshold (PPT) (**B**), muscle soreness at stretching (**C**), and muscle soreness at palpation (**D**).

**Figure 3:**
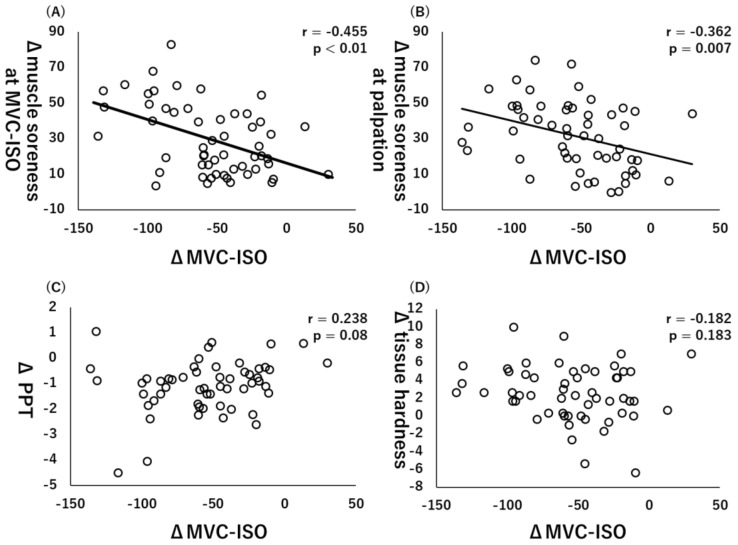
Relationships (Pearson r and *p*-values) between Δmaximal voluntary isometric contraction (MVC-ISO) and Δmuscle soreness at MVC-ISO (**A**), Δmuscle soreness at palpation (**B**), Δpain pressure threshold (PPT) (**C**), and Δtissue hardness (**D**).

All variables are at baseline and 48 h after ECC exercise, and Table 2 shows all the Δ values. Moreover, Pearson’s product–moment correlation coefficient showed that there was a significant correlation between Δknee flexion ROM and Δtissue hardness (r = −0.314, *p* = 0.019, Figure 2A), but there were no significant correlations between ΔPPT (r = 0.131, *p* = 0.341, Figure 2B), Δmuscle soreness at stretching (r = 0.104, *p* = 0.448, Figure 2C), and muscle soreness at palpation (r = −0.21, *p* = 0.125, Figure 3D).

Additionally, there were significant collections between ΔMVC-ISO and Δmuscle soreness at MVC-ISO (r = −0.455, *p* < 0.01, Figure 3A) or Δmuscle soreness at palpation (r = −0.362, *p* = 0.007, Figure 3B), whereas there were no significant correlations between ΔMVC-ISO and ΔPPT (r = 0.238, *p* = 0.08, Figure 3C) or Δtissue hardness (r = −0.182, *p* = 0.183, Figure 3D).

**Figure 4:**
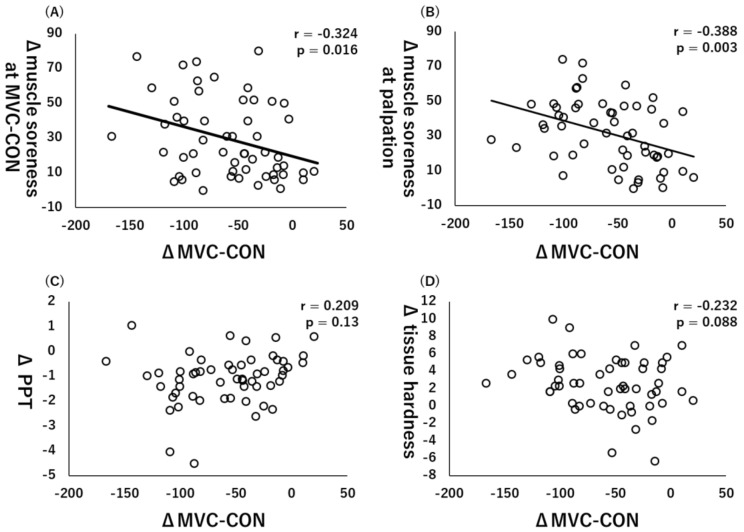
Relationships (Pearson r and *p*-values) between Δmaximal voluntary concentric contraction (MVC-CON) and Δmuscle soreness at MVC-CON (**A**), Δmuscle soreness at palpation (**B**), Δpain pressure threshold (PPT) (**C**), and Δtissue hardness (**D**).

Similarly, there were significant collections between ΔMVC-CON and Δmuscle soreness at MVC-CON (r = −0.324, *p* = 0.016, Figure 4A) or Δmuscle soreness at palpation (r = −0.388, *p* = 0.003, Figure 4B), whereas there were no significant correlations between ΔMVC-CON and ΔPPT (r = 0.209, *p* = 0.13, Figure 4C) or Δtissue hardness (r = −0.232, *p* = 0.088, Figure 4D).

## 4. Discussion

This study investigated the factors associated with decreases in muscle strength and ROM 2 days after ECC exercise when loss of muscle function was assumed to be at the peak. Our results showed a significant association between ROM decrease to the increase in tissue hardness and the muscle strength decrease to muscle soreness. Previous studies investigated the indirect marker of muscle damage after ECC exercise; nevertheless, to the best of our knowledge, this study is the first to identify factors associated with loss of muscle function 2 days after ECC exercise.

Our results showed a significant correlation between ΔROM and Δtissue hardness (r = −0.314, *p* = 0.019). Previous studies showed that tissue hardness and the sensation of the subject are related to ROM in nondamaged muscles [19,20]. Since the increase in tissue hardness could decrease muscle extensibility, this increase in tissue hardness may have led to the decrease in ROM caused by the ECC exercise. Conversely, there was no significant correlation between the change in muscle soreness at stretching and the decrease in ROM (r = 0.104, *p* = 0.448). Previous studies have reported that stretching perception by the subject is related to ROM [21,22]. The discrepancy between the results of this study and those of previous studies is thought to be related to the state of the muscle being measured. In the previous studies, which stated that the sensation of the subject was related, the target was a nondamaged muscle, whereas, in this study, the target was an eccentrically damaged muscle. Additionally, since this study measured maximal knee flexion ROM, the relationship with muscle soreness at stretching might not have been observed.

Muscle soreness at MVC-ISO or MVC-CON was a factor associated with both MVC-ISO and MVC-CON decreases. However, a previous study reported that muscle soreness did not correlate with CK activity (i.e., index of muscle damage) [23]. Moreover, another study reported that muscle soreness caused by ECC exercises might decrease muscle power output [24]. Thus, it is thought that the subjects who experienced a large amount of pain caused by ECC exercises had severe inhibition of muscle power output. In the present study, however, muscle soreness was associated with loss of muscle strength, which likely cannot be explained by the CK activity rather by microdamage of the muscle [1,2].

This study showed that the decrease in ROM caused by ECC exercise was associated with an increase in tissue hardness, and the decrease in muscle strength was associated with muscle soreness. As described above, the ECC emphasized a larger muscle strength increase and muscle hypertrophy when compared with other types of training [3,4,5]. Nevertheless, acutely and up to the time that the muscle is fully recovered, ECC exercise could negatively affect athletic performance, reduce training quality, adherence to resistance, and likely cause higher injury prevalence. Thus, there is a need to speed up the recovery to overcome these side effects. Specifically, static stretching and hold–relax stretching decrease passive stiffness [25]. Additionally, foam rolling intervention decreases muscle pain, including pain threshold [12,13]. Thus, a stretching intervention might effectively counteract the ROM decrease caused by ECC exercise. Moreover, a foam rolling intervention might effectively counteract muscle strength loss. Static stretching intervention for eccentrically damaged muscles increased ROM and decreased passive stiffness [24], and foam rolling intervention improved muscle strength [11]. Future studies should aim to combine stretching with foam rolling [26] to investigate if both ROM and muscle strength can be increased in an eccentrically damaged muscle.

This study did not measure muscle stiffness, but tissue hardness, using the portable tissue hardness meter. The tissue hardness could be affected by the target muscle stiffness (e.g., rectus femoris muscle), as well as the skin, subcutaneous fat, and even deeper muscle stiffness (e.g., vastus intermedius). Thus, future studies should investigate the relationship between ROM or muscle strength changes and changes in muscle stiffness by using, for example, elastography.

## 5. Conclusions

In conclusion, we investigated the factors associated with loss of muscle strength and ROM 2 days after ECC exercise in a large sample (*N* = 56). Our results showed that the ROM decrease could be related to increased tissue hardness, and MVC-ISO and MVC-CON decreases could be related to a muscle soreness increase. Our results suggested that tissue hardness must be controlled for ROM loss, and muscle soreness must be controlled for muscle strength loss.

## Figures and Tables

**Table 1:** The test and retest reliability of knee flexion range of motion (DF ROM), maximal voluntary isometric contraction torque of plantar flexors (MVC-ISO), maximal voluntary concentric contraction torque (MVC-CON), muscle soreness at stretching, MVC-ISO, MVC-CON, palpation, pain-pressure threshold (PPT), and tissue hardness.

	ICC (1, 1)	CV (%)
Knee flexion ROM (°)	0.825	1.6 ± 1.3
MVC-ISO (Nm)	0.928	2.8 ± 1.6
MVC-CON (Nm)	0.958	2.3 ± 1.3
Muscle soreness		
At stretching (mm)	0.987	4.9 ± 3.9
At MVC-ISO (mm)	0.946	5.5 ± 3.9
At MVC-CON (mm)	0.733	5.5 ± 3.3
At palpation (mm)	0.983	4.9 ± 1.8
PPT (kg)	0.966	7.9 ± 5.5
Tissue hardness (N)	0.838	5.3 ± 2.6

CV, coefficient variation; ICC, intraclass correlation coefficient.

**Table 2:** Changes (mean ± SD) in knee flexion range of motion (ROM), maximal voluntary isometric contraction torque of knee extensor (MVC-ISO), maximal voluntary concentric contraction torque (MVC-CON) at 60°/s, muscle soreness at stretching, MVC-ISO, MVC-CON, palpation, pain-pressure threshold (PPT), and tissue hardness before (baseline) and 48-h after maximal eccentric contraction exercise.

	Baseline	48 h after ECC Exercise	Δ Change from Baseline to 48 h after ECC Exercise
Knee flexion ROM (°)	136.9 ± 6.7	116.5 ± 16.9	−20.4 ± 16.1
MVC-ISO (Nm)	159.4 ± 26.0	104.5 ± 32.0	−54.8 ± 36.0
MVC-CON (Nm)	166.4 ± 27.1	107.9 ± 38.6	−58.5 ± 42.4
Muscle soreness			
At stretching (mm)	5.5 ± 13.2	39.2 ± 22.1	33.7 ± 20.3
At MVC-ISO (mm)	6.6 ± 8.5	36.4 ± 20.9	29.7 ± 19.4
At MVC-CON (mm)	6.2 ± 8.5	35.6 ± 22.6	29.3 ± 22.2
At palpation (mm)	10.3 ± 9.8	42.6 ± 18.5	31.7 ± 18.8
PPT (kg)	2.6 ± 1.2	1.5 ± 1.0	−1.1 ± 1.0
Tissue hardness (N)	18.0 ± 4.0	20.5 ± 3.8	2.6 ± 3.1

## Data Availability

All data supporting the conclusions of this study will be fully provided upon request by authors.
